# Advancing engineering research through context-aware and knowledge graph–based retrieval-augmented generation

**DOI:** 10.3389/frai.2025.1697169

**Published:** 2025-11-19

**Authors:** Soham Ghosh, Gaurav Mittal

**Affiliations:** 1 Department of Electrical Engineering, Black & Veatch, Overland Park, KS, United States; 2 IEEE IAS/PES Kansas City Section, Kansas City, Kansas,United States; 3 Enterprise Solutions Architect, Black & Veatch, Overland Park, KS, United States

**Keywords:** context-aware information retrieval, RAG architectures, engineering design automation, knowledge graphs, LLM and intelligence

## Abstract

Large language models (LLMs) are powerful in language understanding and content generation but frequently fall short of technical accuracy when they are applied to engineering code, standards, and design documents. To mitigate this, we are seeing the emergence of Retrieval-Augmented Generation (RAG) models that ground outputs of LLMs with information from external trustworthy resources, increasing the factual consistency. However, traditional RAG techniques are limited in the treatment of isolated information (limited to the amount of information in a fixed-size chunk) and are deemed ill-equipped to traverse semantically linked technical information. This study introduces a collection of new and highly deployable RAG-LLMs built on the n8n automation system and specifically designed for engineering domains. Framework effectiveness was tested on a set of prompts developed with the help of practicing electrical engineering professionals and should be read through the framework’s lens for interpretation of national engineering codes, technical standards, and design standards. To mitigate the shortcomings of the conventional retrieval-based chunking methods, a contextual RAG-based approach is employed to align the retrieved content with the query context to improve relevance. Moreover, RAG is adopted to structure knowledge graph retrieval, which can retrieve densely linked concepts from multiple knowledge graphs, thereby promoting more profound semantic understanding in complex technical domains. The study describes the relative benefits of these improvements, points to practical deployment issues, strengths, and weaknesses. All the n8n workflows employed in this study are made available as supplementary materials to facilitate reproducibility and sharing within the engineering research community and practitioners.

## 1 Introduction

Over the past half a decade, large language models (LLMs) have revolutionized many fields, ranging from natural language processing to artificial intelligence. However, despite their extraordinary linguistic skills, LLMs are known to be highly prone to hallucinations, i.e., outputs that are factually wrong or non-sensible for no evident reason. This is due to several challenges of LLM design and operation, which include but are not limited to the use of large training datasets that are often unfiltered, and to the auto-regressive nature of these models, which can cause generated content to be inconsistent (Li et al., 2023; Roustan and Bastardot, 2025). As such, LLM hallucinations can take forms such as fake evidence, false inference, and totally wrong replies. These hallucinations can be dangerous in critical applications like engineering, reasoning, and diagnosis, and information retrieval (Zhang et al., 2023). For example, one study shows that the hallucination rate for the different LLMs ranges between 50 and 82% depending on the operational settings, emphasizing the prevalence of such a phenomenon in machine-produced texts (Omar et al., 2025).

Because hallucinations can have severe consequences, particularly in fields like engineering, healthcare, and law, there is a pressing need to explore new approaches that make LLM outputs more dependable. One approach with potential is Retrieval Augmented Generation (RAG), which integrates LLMs’ skills with retrieval-based approaches to enhance correctness and relevance. RAG enables LLMs to dynamically and modularly include external sources into the generation flow, which greatly mitigates the limitation of being confined to pre-defined training datasets (Gopi et al., 2024; Siddharth and Luo, 2024). In an applied context, leveraging RAG technology is observed to significantly enhance the capabilities of LLMs in emergent scenarios such as rapid identification of relevant clauses in engineering design code and standard or query-based knowledge exploration for an engineering document cluster. By allowing LLMs to access additional information, a RAG system increases coherence and factuality of outputs, demonstrating their wide applicability across different high-stakes settings (Superbi et al., 2024).

In addition, the recent developments in the comprehension and management of hallucinations relate to the increase in the use of RAG techniques. Using RAG mechanisms (which mix generative capabilities with well-defined retrieval processes), LLMs can self-repair incorrect results by referencing (on-the-fly access) information fetched dynamically from reliable databases. This not only provides the means for a more dependable computational environment but also enhances user confidence in intelligent systems (Chen et al., 2023). As LLMs develop, the integration of RAG methods seems to be an important move in order to guarantee that such technologies are capable of generating suitable, context-sensitive, and reliable answers in a growing variety of use cases. Although retrieval-augmented generation (RAG) models can address the aforementioned problems by grounding answers in well-documented sources, the actual implementations of RAG for engineering remain underdeveloped, and there are a number of practical challenges hindering their implementation. These are as follows:

1. The barrier to entry is still quite high for the RAG-LLM combination, given that building a well-performing RAG-LLM pipeline usually demands a good deal of programming expertise, which may not be available to many engineering professionals and researchers.
2. For an RAG-LLM application to truly be successful, it should have the ability to access a user’s existing document repository in a manner similar to popular applications like Google Drive or SharePoint. Unfortunately, building such integrations is never that simple, and would likely add layers of complexity, such as us having to implement authentication protocols, configure a set of APIs, and understand how to parse a document.
3. In order to make a RAG pipeline work well, one often also has to adopt more advanced tweaks, such as reranking strategies, context retrieval mechanisms, or knowledge graph-based augmentation. While those approaches increase retrieval precision and relevance of output, they tend to be technically challenging and are based on the use of several NLP tools and frameworks. Altogether, these subtleties limit the accessibility and effective usage of RAG-LLM systems for numerous engineers and applied researchers.

Real-world engineering research and application have faced these obstacles down, and many practitioners have included them as “given” variables. To mitigate the high entry barrier and technical complexities, a fast-deployable recipe has been articulated employing open LLMs and modular RAG pipelines and enjoying simple deployment capabilities at accessible platforms such as LM Studio, AnythingLLM, and n8n. This eliminates the requirement for elaborate programming and allows practitioners to remain focused on the engineering content and not the software infrastructure. A number of workflow implementations have been showcased in the n8n automation framework, including document retrieval, reranking, contextual querying, and knowledge graph integration. Performance testing has been performed to a large extent, based on engineering documents, technical standards, and literature from, e.g., the National Fire Protection Association (NFPA), the Institute of Electrical and Electronics Engineers (IEEE), and the International Council on Large Electric Systems (CIGRE). The findings validate that a rapidly deployable RAG-LLM design can achieve good usability and high performance in technically challenging domains.

### 1.1 Manuscript contribution

This study pushes forward the methodology when it comes to the development of rapidly deployable retrieval-augmented generation (RAG) systems and evaluation of technical document querying tuned for practicing power system professionals. The specific contributions are as follows:

1. *RAG-LLM pipeline implementation:* Example of a rapid deployable RAG-LLM pipeline, using LM Studio and AnythingLLM to secure local document retrieval and language model inference is provided. The code to be used for parsing and embedding corpus-specific documents is released and available through the ‘Data Availability’ section of the article.
2. *Practitioner-oriented evaluation and design considerations:* The manuscript frames the design and evaluation of RAG workflows with a focus on practitioner guidance, particularly for electrical engineers and applied researchers working in rapidly deployable environments such as n8n. Special attention is given to the challenges encountered by traditional RAG systems when processing dense tabular data and multi-layered exceptions commonly found in technical codes and standards.
3. *Contextual retrieval workflow via n8n:* We introduce a novel RAG contextual workflow implemented in n8n, which supports better processing of tabular structures and ‘exception logic’ by orchestration of document processing and dynamic retrieval logic. The full workflow is released to the public and is available through ‘Data Availability’ for reproducibility and the enablement of practical use.
4. *Knowledge graph-based retrieval workflow via n8n and Infranodus:* We present a state-of-the-art knowledge graph-augmented RAG workflow leveraging n8n and Infranodus to semantically structure engineering documents and reveal hidden relationships among engineering concepts. This method allows developers to make exploratory queries and cross-reference between linked standards, which is beneficial for complex design and compliance activities. The full process, from graph construction to integration and retrieval logic, is released to the public in the ‘Data Availability’ section for reproducibility and to serve as a practical tool.

### 1.2 Manuscript organization

The remainder of the manuscript is structured as follows: in Section 2, we describe the basics of Retrieval Augmented Generation (RAG), which presents the basic building blocks of the RAG system and a typical structure of the RAG system architecture. Section 3 describes a simple-to-adopt private RAG framework constructed from open-source LLMs, which uses LM Studio and AnythingLLM to be deployed locally. In Section 4, we focused on quickly deployable integration of n8n for document retrieval, performing an in-depth evaluation and ranked prompts based on a question set built from the NFPA 70 National Electrical Code (NEC) 2017. This section also highlights important observations about when traditional RAG-LLM-based pipelines do and do not perform well. Section 5 mitigates the above restrictions via targeted improvements in the area of reranking and contextual RAG workflows. We show scores in comparison to various rerankers (e.g., bge-reranker-base and Cohere Reranker v3. 5) over various embedding models such as OpenAI and Voyage. As an advanced use case, Section 6 discusses a multi-brain RAG implementation of a state-of-the-art knowledge graph-based RAG system and how it can be applied for exploratory querying on a network of engineering standards. Practical guidelines for reducing the consumption and related costs of APIs are presented. Finally, Section 7 summarizes with a summary of our findings, the current limitations, and future work ahead.

## 2 Fundamentals of retrieval-augmented generation (RAG) and present limitations

Large language models (LLMs), such as GPT-4 and LLaMA, have demonstrated remarkable capabilities at generating coherent, contextually appropriate text. They have an impressive flaw, though they often “hallucinate,” generating plausible-sounding but incorrect (or impossible to verify) information. This observation is due to the fact that LLMs produce outputs by relying on statistical associations in their training data and not on grounded truth or domain-specific knowledge. To address this limitation, retrieval-augmented generation (RAG), a novel architectural style, has emerged as a compelling approach. With RAG, the generation process is combined with a retrieval module to dynamically retrieve relevant documents or context passages from an external knowledge source. This is similar in spirit to giving a model access to a domain-specific textbook at test time, to consult ground truth sources before generating answers.

**Figure 1:**
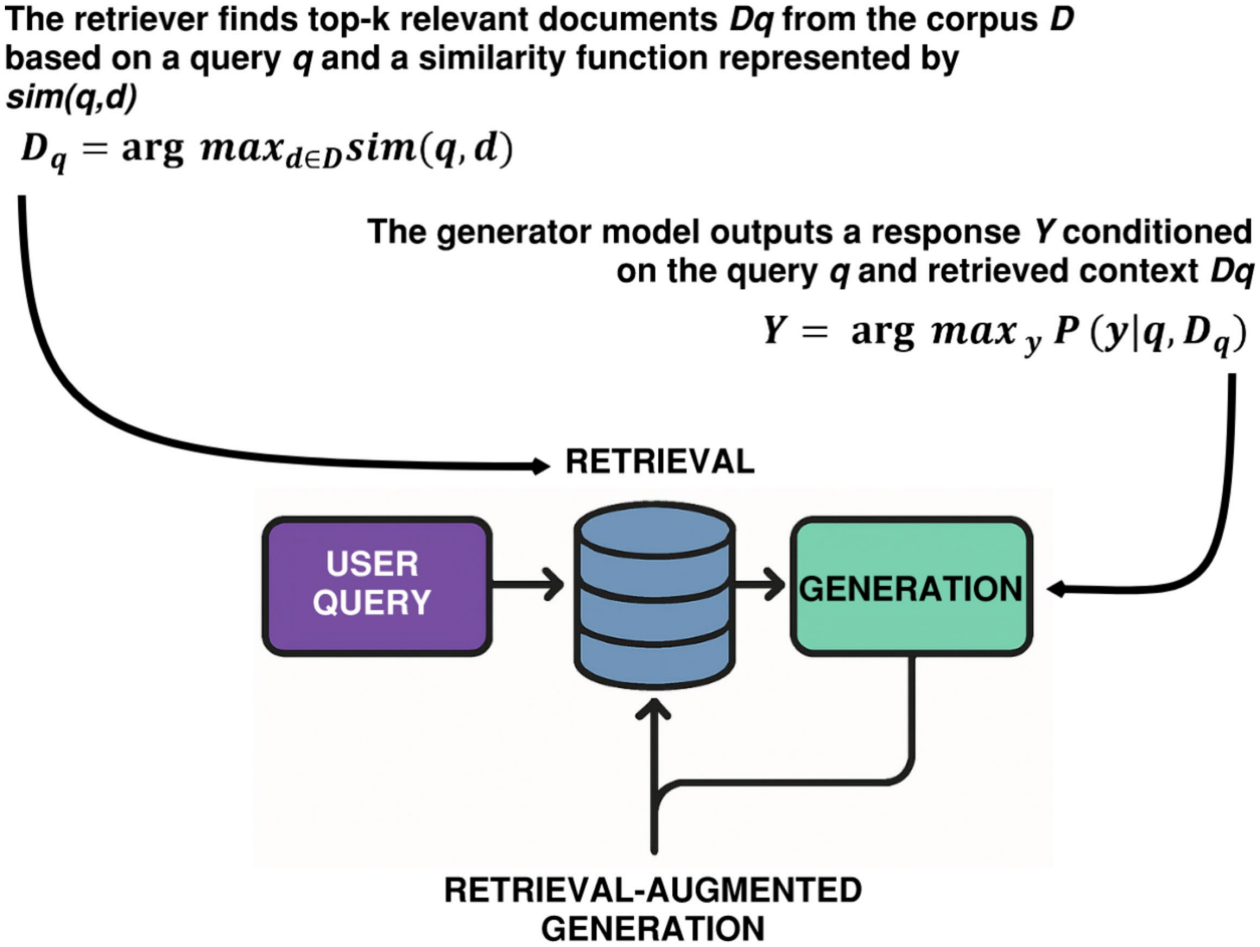
Illustration of RAG architecture, where a user query initiates a retrieval process from a knowledge base (e.g., vector database or document store).

By building retrieval directly into the generation loop, as illustrated in Figure 1, a RAG system can largely improve the end quality of correctness and domain relevance. For example, in power systems engineering or regulatory compliance, where specific terms and references are required, RAG provides answers that are tied to source materials collected for that domain. This renders RAG especially well-suited for use cases where there is a high emphasis on knowledge-intensive question answering, code documentation, engineering reasoning, and queries, and where hallucinations can be high-stakes. Nevertheless, RAG is not a replacement for domain adaptation or continual learning. It does not allow a model to generalize new syntactic constructions, programming languages, or stylistic conventions. Instead, it offers an efficient amplification process for enhancing response accuracy within a predefined information domain. In this way, RAG facilitates retrieval, and not representation, which is more suitable for fine-grained applications rather than general domain training.

Although hosted LLM interfaces like ChatGPT offer the ability for a user to upload domain-specific documents (e.g., an electronic engineering textbook or an IEEE master volume) and then engage in retrieval-augmented querying, there are multiple limitations when one has to resort to a cloud-based service for professional or industrial use. These are as follows:

1. Data sovereignty and confidentiality, become a pressing concern. Uploaded documents are outside of the control of personal or corporate workstations, effectively by-passing internal policies on data handling, intellectual property agreements, or even regulatory requirements, especially when proprietary designs, sensitive specs, or restricted standards are concerned.
2. Hosted models often tend to have limits on the length of tokens and contexts that make it hard for technology to work. For instance, in the case of a long publication such as a multi-chapter standard or a big handbook, the material may go above the maximum token limit, even with approaches for breaking up and summarizing documents. This might lead to insufficient context or retrieval and may make response generation less accurate.
3. In the case of hosted solutions, there is usually no persistent user-manageable storage of the parsed knowledge. After the document is uploaded and used for one session, the embedding and index layers are transient and not seen (allowed access to) by the user. This makes it impossible for engineers to construct long-term, reusable vector databases that can adapt to the progress of ongoing projects or to organizational requirements in terms of the knowledge needed.
4. The other limitation is the higher cost of operation, and this is more applicable to frequent or large-scale document processing. Subscription fees, usage-based pricing, and data upload limitations while attempting to submit jobs to external services can add up to prevent hosted LLM services at scale economically feasible for long-term engineering workflows or enterprise integration.

These shortcomings also reveal the importance of self-hosted RAG pipelines, i.e., a system where people could download LLMs themselves and run them locally, parse their own engineering documents, and keep the embeddings in a vector database of their selection (e.g., FAISS, Pinecone, or Qdrant). This kind of configuration gives users full access to the retrieval layer, the ability to customize indexing strategies, and integrate with in-house tools or platforms. In the context of electrical engineering, this means sensitive design documents, grid planning standards, or equipment protocols can be queried securely, reliably, and repeatedly, without using third-party APIs or external cloud services. It also provides the opportunity for sensitive document access policies, auditability, and retention of understanding over time, which is important in high-assurance engineering contexts.

## 3 Opensource LLM and private RAG-LLM pipeline for engineering applications

Open-source large language models (LLMs) are especially attractive in that their deployments can be conducted locally on premises, such that users can locally perform inference in a secure and private way and without relying on proprietary API endpoints or any external, possibly cloud-based, service. This is especially useful in retrieval-augmented generation (RAG) tasks in engineering domains where data privacy, reproducibility, and system-level control are important. Engineers can consult the Hugging Face Open LLM Leaderboard (Huggingface, 2025) to find candidate models for local deployment. Through the filtering into “mid-range” models (typically 14B-32B range as shown in Table 1), users can choose those models that compromise between inference performance and hardware feasibility.

**Table 1:** Open LLM leaderboard filtered by ‘mid-range’ models and sorted by weighted average of normalized scores from all benchmarks (as of June 2025).

Model	Average score across all benchmarks	IFEva^†^	GPQA^‡^	CO_2_ cost
Qwen2.5-test-32b-it	**47.37%**	78.89%	15.21%	29.54 kg
Horizon-AI-Avengers-V1-32B	47.34%	79.72%	14.99%	7.95 kg
FluentlyLM-Prinum	47.22%	80.90%	18.23%	21.25 kg
Qwen.5-14b-v1.0-e3	47.09%	73.24%	22.26%	**1.56 kg**
Qwen2.5-32B-Instruct-abliterated-v2	46.89%	83.34%	15.66%	13.49 kg

^†^Instruction-following evaluation (IFEval): This metric tests the model’s ability to follow explicit formatting instructions.

^‡^Graduate-level Google-Proof Q&A (GPQA): This metric scores the LLM performance based on PhD-level knowledge multiple-choice questions in science.

Boldface scores represent the highest scores or the lowest carbon footprint.

**Figure 2:**
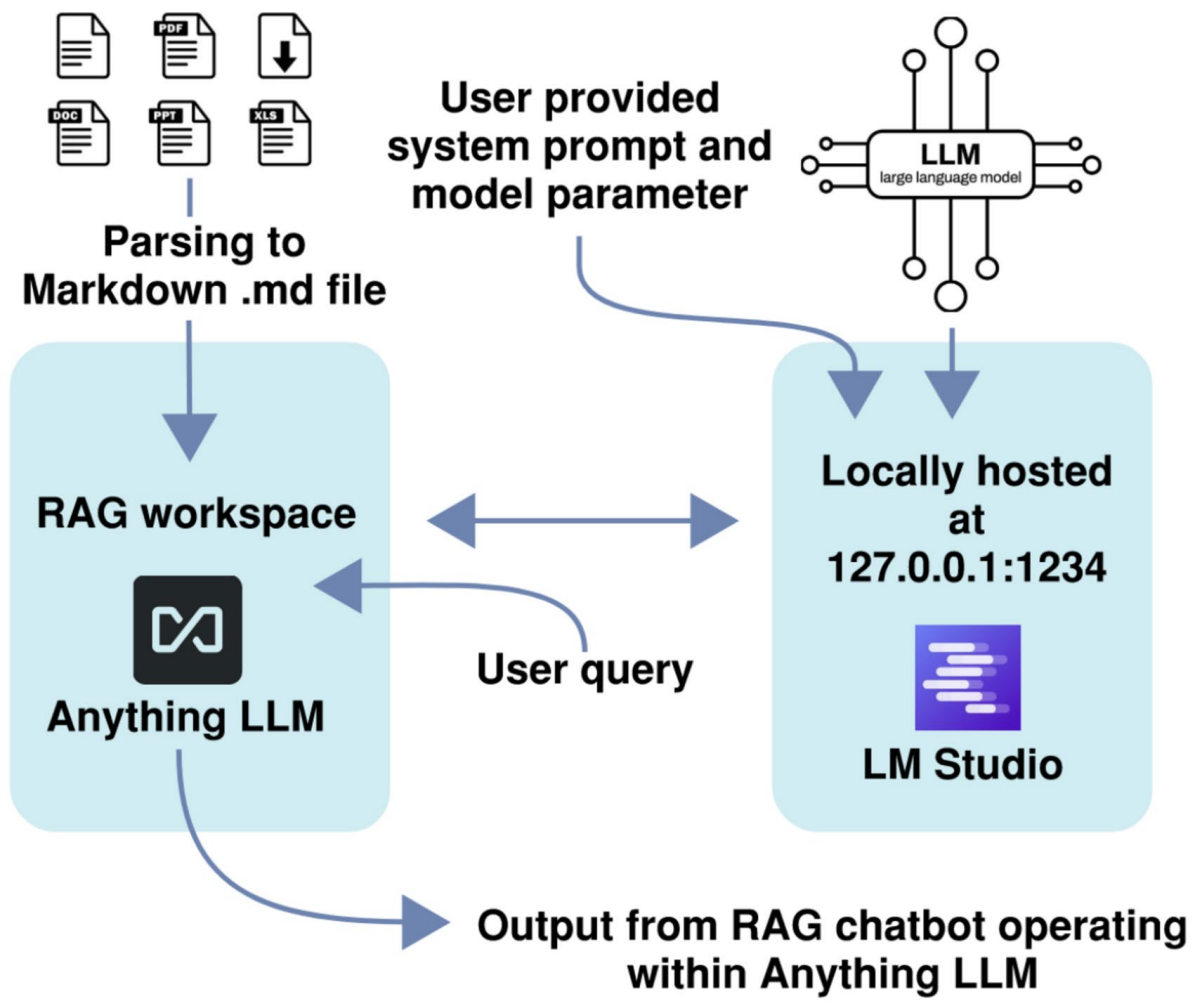
Illustration of a private and locally executed RAG-LLM pipeline using LM Studio and AnythingLLM.

The private and locally executed RAG-LLM leverages two open source softwares, namely LM Studio and AnythingLLM; see Figure 2. Within LM Studio, users can download target LLM models from open source and launch them in their quantized formats (e.g., GGUF) without extra tuning. The settings that may be tuned are those of the system prompt, randomness temperature, and top-K sampling parameter. For an entire RAG pipeline, a second open-source application, AnythingLLM, is leveraged for establishing a local retrieval work environment. PDF files consisting of IEEE standards, electrical codes, or equipment manuals have to be initially changed into the Markdown (. md) and one may upload them to AnythingLLM. Markdown works better for RAG because it provides clean, structured, and token-efficient text that enables accurate chunking and retrieval, unlike the noisy and layout-heavy content in PDFs.

AnythingLLM also permits users to set the ‘Text Chunk Size’ and ‘Text Chunk Overlap’, which are crucial parameters that determine how fine and how much of the context is preserved in document embeddings. AnythingLLM communicates with LM Studio (which is (supposed to be) running locally), in which we embed the LLM, and it makes requests to the cached model to generate text in response to questions about the embedded content. Such a setup guarantees that the model and the knowledge base are local to the user’s computer, and that the user remains in complete control of the data, the execution, and the experimental reproducibility, without the need to rely on cloud inference or third-party services.

## 4 Rapidly deployable integration with n8n for document retrieval and framework limitations

Although LM Studio and AnythingLLM provide a fast, secure, and locally deployable solution for developing retrieval-augmented generation (RAG) systems, it comes with limitations in workflow automation, integration, and orchestration capability. For more advanced use cases, when integrating document ingestion, vector store, and even multi-channel query, a flexible, growable automation frame is required. Which is precisely where n8n, a free and open-source, node.js-based workflow automation tool, excels. User of n8n can create automated workflows that incorporate AI with corporate-level business process automation. It runs on-premises, which guarantees data privacy and allows for custom logic, APIs, and plugins, so it is appropriate for engineering, document intelligence, and RAG applications.

**Figure 3:**
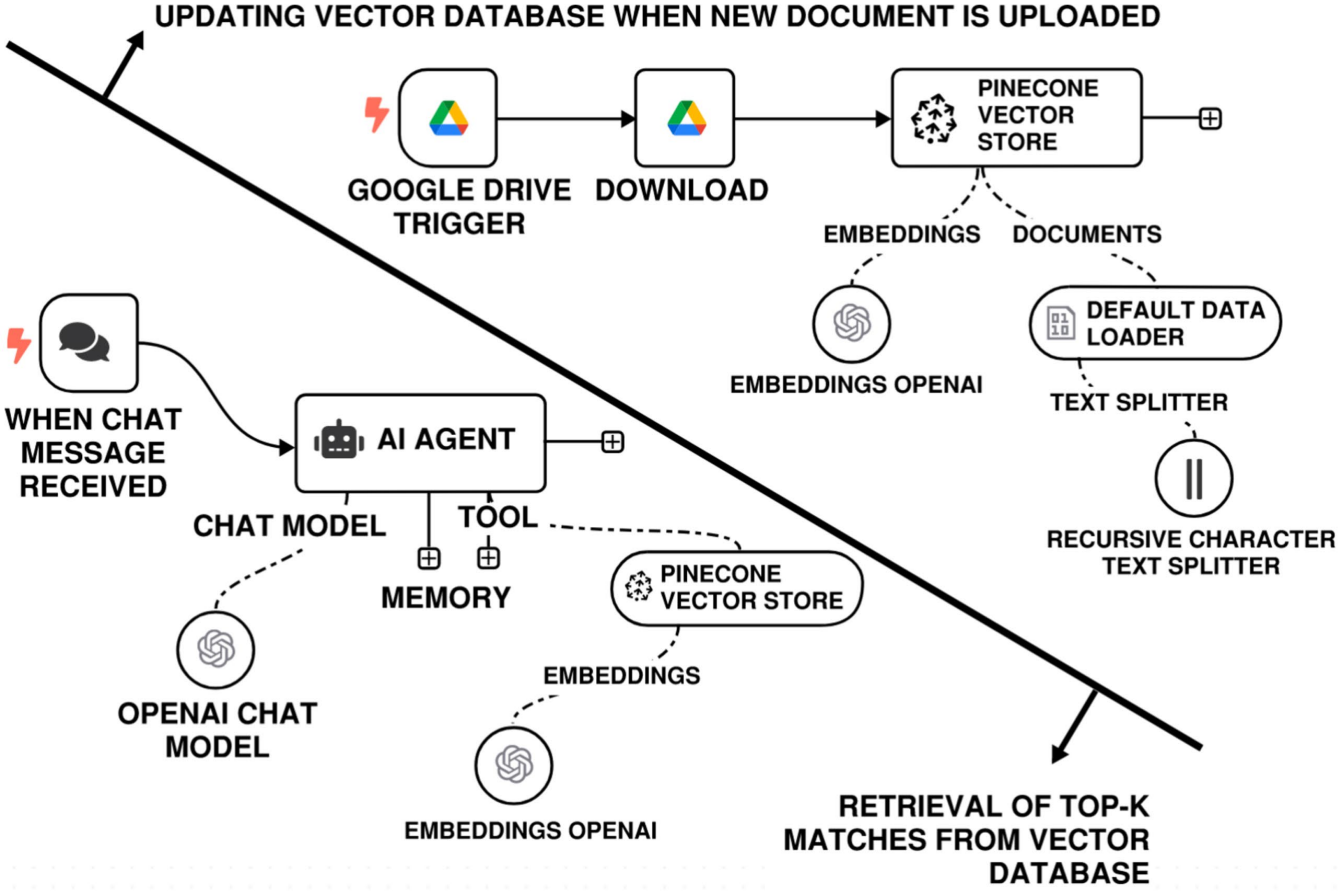
n8n RAG-LLM workflow with a Google Drive-based retrieval system offering a user-friendly framework for engineers and researchers.

Figure 3 shows an example RAG workflow using n8n based on the use case of this paper. The workflow functions as follows:

1. *Document upload:* Engineers/technical professionals upload documents such as PPT, PDF, Datasheets, Word-based Specifications, or Manuals built as per the standards in 1 above, into a Google Drive folder assigned for them.
2. *Automated ingestion:* n8n monitors the Google Drive folder for new uploads. When a file is discovered, it initiates a processing pipeline to extract the content, optionally parse it into Markdown or plain text, and embed the text using the user-provided embedding model.
3. *Vector storage:* The resulting representations are saved in a vector database (can be local or cloud-hosted), such as Qdrant, Chroma, Pinecone, to be queried against later.
4. *Query interface:* The end users pose queries using different interfaces such as Gmail, Slack, or a web-based chatbot. There, an n8n agent returns the top-K related chunks from the vector database.
5. *LLM generation:* The resulting snippets are sent to the LLM of the user’s preference1, which is hosted in a local machine to generate a grounded, context-aware reply.

This modular and event-driven design further decouples ingestion and querying phases, and allows users to automate multi-step, cross-platform knowledge workflows, resulting in a more scalable and maintainable RAG-LLM solution in engineering settings.

In order to demonstrate the effectiveness of the proposed RAG-LLM pipeline, we performed an experiment on a collection of curated, domain-specific search queries to the National Electrical Code (NEC), a complex regulatory document relied on by practicing electrical engineers. The NEC codebook (in parsed text) was uploaded by the user to the RAG system, allowing for grounded-document retrieval for inference. The evaluation searches were specified to check if the system can:

1. Locate and extract numerical values from NEC tables (e.g., minimum burial depths, conductor ampacity ratings, and overcurrent protection limits).
2. Extract exceptions and conditional clauses from the codebook, as they frequently manifest as annotations or as structural deviations from the norm.
3. Retrieve the relevant sections and apply necessary contextual logic (e.g., identifying requirements that vary by installation type, voltage class, or application environment).

Each generated response was evaluated against user-defined ground truth answers, see Table 2, simulating expert validation. For scoring, we used the correctness measure of DeepEval with GEval criteria, which assesses semantic alignment, factual grounding, and contextual completeness between the model’s response and the reference answer.

**Table 2:** Validation of traditional RAG-LLM responses using an n8n workflow on query tasks based on the 2017 National Electrical Code (NEC).

Question and ground truth	Actual output and evaluation
**Q1. Prompt:** I am trying to find the table in the NEC that deals with a single-phase AC motor and the associated full load current. **A1. Ground truth:** Table 430.248 of the NEC addresses single-phase AC motors. It also addresses the corresponding full load current.	**A1. Actual output:** The NEC section is Article 430. This section is titled “Motors, Motor Circuits, and Controllers.” Do refer to Table 430.248 of Article 430 to determine the minimum full-load current required for a single-phase AC motor, as indicated by the motor’s nameplate rating. **Score: 0.9072185128712421** **Reason:** The output was found to correctly cite NEC Table 430.248. It aligns with the expected outcome. It additionally provides supplementary context by citing Article 430 and its title, both of which augment factual accuracy and comprehensiveness. The supplementary detail slightly exceeds the expected simplicity; yet, there are no discrepancies or omissions.
**Q2. Prompt:** I want to know what is the minimum cover required per Section 300.5 of the NEC, for direct buried cables running under a parking lot, based on Table 300.5. **A2. Ground truth:** According to Table 300.5 of the NEC, the minimum cover necessary for a direct buried cable beneath a parking lot is 24 inches.	**A2. Actual output:** According to NEC Table 300.5, the minimum burial depth for a direct-buried cable installation under a parking lot is 24 inches (609 mm). This relates to the upper surface of the finished grade, and further criteria may be considered when determining the appropriate cover for your specific installation. **Score: 0.859266659995407** **Reason:** The actual production accurately determines the necessary minimum cover as 24 inches, consistent with the anticipated output. It offers supplementary context by specifying the measurement in millimeters and acknowledging that other factors may affect the cover, which, although not included in the anticipated output, does not contradict it. The information is accurate and complete; nonetheless, the additional details somewhat diverge from the anticipated conciseness.
**Q3. Prompt:** In the NEC, which section deals with the minimum cover requirement for cables and conduits. **A3. Ground truth:** In the NEC, section 300.5 deals with the minimum cover requirement for cables and conduits.	**A3. Actual output:** The minimum cover requirements for cables and conduits are specified in Section 300.5 of the NEC. This section delineates the minimum cover requirements for direct-buried cables, conduits, or other raceways. Furthermore, additional information concerning mechanical protection for direct-buried cables can be found in sections 840.48 and 830.47 of the NEC. **Score: 0.8009300906486064** **Reason:** The output accurately identifies Section 300.5 of the NEC as pertaining to minimum cover requirements for cables and conduits. It also offers further information regarding related areas, which is not mandatory but does not contradict the anticipated outcome. There are no factual flaws or omissions about the basic topic, but the other facts, albeit accurate, are superfluous to the specific inquiry.
**Q4. Prompt:** Is there any restriction on running conductors in parallel in the NEC. **A4. Ground truth**: NEC section 310.10(H) talks about conductors in parallel.	**A4. Actual output:** Indeed, Section 310.10(H) of the NEC permits the installation of parallel conductors in raceways, multiconductor cables, or direct-buried single-conductor cables. **Score: 0.5432697039021899** **Reason:** The actual output accurately recognizes NEC section 310.10(H) as pertinent to parallel conductors, consistent with the anticipated output. However, the output introduces supplementary details regarding installation methods that are not in the anticipated output. These additional outputs may be perceived as superfluous information. While there are no factual flaws or contradictions, the additional details may detract from the emphasis on the essential information.
**Q5. Prompt:** Based on section 310.10(H) of the NEC, if I have two parallel #8 AWG conductors, what is my restriction on overcurrent protection? **A5. Ground truth:** The overcurrent protection for two parallel #8 AWG conductors must ensure that the ampacity of each individual conductor is not surpassed if one or more of the parallel conductors become unintentionally disconnected.	**A5. Actual output:** Section 310.10(H) of the NEC permits parallel conductors in raceways, multiconductor cables, or direct-buried single conductor cables, provided they satisfy specific criteria: One of these is that the cumulative current-carrying capacity of the parallel circuits must not surpass the ampacity rating of the raceway or overhead circuit. In the specified context, when utilizing two parallel #8 AWG conductors, it is crucial to verify that the cumulative current-carrying capability of both circuits does not surpass the ampacity rating of the raceway or overhead circuit. **Score: 0.2610665811531422** **Reason:** The actual output fails to specifically mention the restriction on overcurrent protection related to the scenario where one or more parallel conductors become disconnected, which is a key aspect of the expected output.
**Q6. Prompt:** I am trying to find the table in the NEC that deals with AC motors and associated current. **A6. Ground truth:** Tables 430.248 through 430.250 reflect the typical and most used 4-pole and 2-pole induction motors (AC) in use.	**A6. Actual output:** Article 440 of the National Electrical Code (NEC), entitled “Motor Controllers,” addresses AC motors and their corresponding currents. Refer to Table 440.5 of Article 440 for the minimum full-load current specifications for single-phase or three-phase AC motors, as determined by the motor’s nameplate rating. **Score: 0.20462199541570797** **Reason:** The actual output erroneously designates Article 440 and Table 440.5 as the pertinent part for AC motors. The correct output should point to Tables 430.248 through 430.250. This signifies a factual inaccuracy and a substantial discrepancy.

Score is computed using DeepEval’s correctness with the criteria [GEval] metric.

^*^A parsed version of the 2017 NFPA 70 – National Electric Code (NEC) was used for the above RAG implementation.

The GEval metric provides a detailed evaluation based on the following criteria:

1. *Grounding:* Did the model reference or integrate pertinent language from the retrieved NEC section?
2. *Exactness:* In terms of exactness, was the LLM output able to accurately deduce essential quantitative thresholds and regulatory stipulations?
3. *Verifiability:* Can the assertions made by the LLM output be traced back to the source sections of the document?

This evaluation framework ensures that the RAG-LLM system not only generates plausible text but also provides code-compliant, reference-anchored answers suitable for technical decision-making in real-world engineering workflows.

Several significant insights were derived from the assessment of the RAG-LLM pipeline via NEC-based queries:

1. *Semantic prompting and section identification:* The RAG-LLM workflow demonstrated efficacy in identifying and correlating the relevant NEC regulations for a specified inquiry. The accuracy was enhanced when prompts were subtly reconfigured to more effectively guide the model toward the desired retrieval pathway. Inquiries that reflected the phrasing or technical terminology of the NEC yielded more accurate outcomes, highlighting the importance of semantic alignment between user input and coding language.
2. *Numerical table retrieval and chunking limitations:* The system’s effectiveness in handling numerical lookup queries from tabular data was only somewhat successful. Challenges were observed with NEC tables, where the retrieval procedure often failed to consistently reproduce the table. The main cause for this behavior is in the method of content division: when vast tables are segmented into multiple sections, the model tends to frequently acquire only fragments of the original context. This limited viewpoint may generate responses that are either incorrect or just partially accurate. From the way the table’s continuity is digested, it is recognized that there is a shortcoming in current Retrieval Augmented Generation approaches.
3. *Challenges with multi-condition exceptions:* The RAG-LLM pipeline faced challenges with regulations using multi-conditioned exceptions, commonly seen in NEC provisions that provide different requirements dependent on voltage, environment, or application type. These exceptions are often expressed as enumerated or hierarchical logical criteria, and the system faced difficulties in analyzing and reasoning through the extensive array of situations. As a result, responses sometimes omitted critical qualifying criteria or incorrectly applied the rule entirely. This indicates a current deficiency in handling hierarchical exception logic within extensive regulatory documents.

These observations suggest specific areas for refinement, particularly in document chunking, semantic prompting, and exception reasoning, which point toward the need for more advanced RAG strategies such as reranking based on a higher number of top-K results and contextual RAG strategies. These enhancements are discussed in detail in the subsequent section.

## 5 Novel enhancements to traditional RAG-LLM workflow to improve performance

The following section presents targeted upgrades, including re-ranking and contextual retrieval, to overcome the constraints of the typical RAG-LLM pipeline outlined in the preceding section, specifically addressing issues of relevance and specificity. Although sophisticated, the additional features are easy to integrate into the fast-deployable n8n approach. For the purpose of reproducibility and adoption in practice, these advanced n8n workflow JSON files are made available and referred to in the “Data Availability” section.

### 5.1 Enhancements based on reranking

Reranking is a crucial stage in modern information retrieval and retrieval-augmented generation (RAG) pipelines, where a basic retriever first gathers a diverse set of candidate documents, subsequently followed by a more sophisticated model that rearranges them based on their true relevance to the query. Among reranking approaches, cross-attention-based reranking is notable for its ability to represent intricate semantic congruence between the query and candidate passages. Unlike bi-encoder systems that independently encode queries and documents into fixed-length embeddings, cross-attention models (such as BERT or T5 in a cross-encoder configuration) concurrently process the query-document pair within a cohesive transformer architecture. This allows the model to compute token-level interactions, thereby concentrating on contextually relevant segments of each passage in relation to the query. As a result, the representations yield significantly more accurate relevance ratings, often leading to a considerable enhancement in retrieval performance, especially in tasks requiring nuanced understanding or disambiguation. This method requires heightened processing capabilities, making it relatively unfeasible for initial retrieval from large data sets; nonetheless, it is exceptionally effective for reranking a limited selection of top-K candidates, attaining a robust balance between precision and scalability.

**Figure 4:**
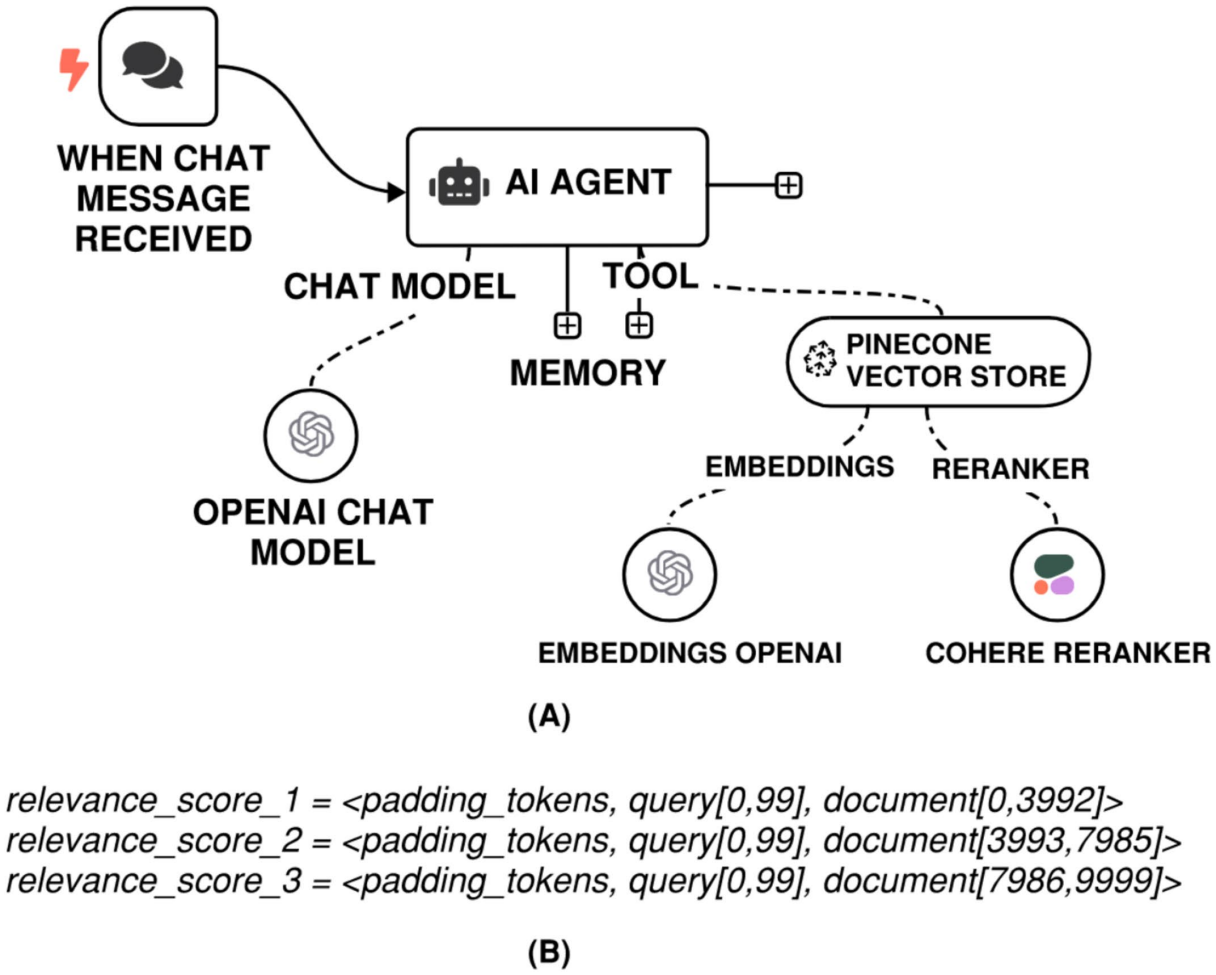
**(A)** n8n RAG-LLM workflow with Cohere reranking. **(B)** Relevant scoring based on Cohere chunking on a 10,000-token document.

On the implementation side, reranking-based gains are within reach in an n8n pipeline. A vector database in n8n could be enabled to rerank results and link the reranking node to a reranking model, such as Cohere Rerank 3.5, see Figure 4A. At the back-end, the Rerank API disaggregates the input query into smaller text segments according to the relevant document. Each segment comprises the query followed by a portion of the document, with the segment size determined by the context length of the employed model. For instance, contemplate the subsequent scenario:

- The model in use is rerank-v3.5, which supports a maximum context length of 4,096 tokens, and
- The input query consists of 100 tokens, and,
- The document to be ranked is 10,000 tokens long, and
- Document truncation is turned off by assigning ‘max_tokens_per_doc’ a value of 10,000.

Under this setup, the API splits the document into three chunks, as illustrated in Figure 4B. The final relevance score assigned to the document corresponds to the highest score obtained across these chunks.

To assess the performance of various embedding models and rerankers, multiple retrieval tests were conducted. Table 3 presents the evaluation results using two standard information retrieval metrics: hit rate and Mean Reciprocal Rank (MRR). Three reranking configurations were compared:

1. Without any reranking (baseline),
2. bge-reranker-base, and
3. Cohere V3.5 reranker.

**Table 3:** Evaluation of rerankers using two standard information retrieval metrics.

Embedding	Without any reranking (baseline)		bge-reranker-base		Cohere V3.5 reranker	
	Hit rate	MRR	Hit rate	MRR	Hit Rate	MRR
OpenAI	0.828	0.692	0.899	0.822	**0.932**	**0.877**
Voyage	0.766	0.650	0.818	0.777	0.891	0.830
Google-PaLM	0.815	0.676	0.892	0.752	0.916	0.865

Boldface score indicates the highest performance.

With results documented in Table 3, the following conclusions were reached:

1. OpenAI + Cohere rerank combinations were found to consistently achieve the highest scores across both hit rate and MRR, thereby positioning them as the top-performing setup.
2. CohereRerank and bge-reranker-base provide consistent enhancements across various embedding models. This demonstrates their strength and effectiveness in improving search quality, irrespective of the type of embedding backbone employed.

The influence of rerankers cannot be overstated, as they play a key role in improving the MRR for many embeddings, showing their importance in making search results better. In the next section, the discussion will be directed toward another popular RAG enhancement technique using contextual retrieval.

### 5.2 Enhancements based on contextual retrieval pipeline and its advantages

For traditional RAG pipelines, source documents are split into smaller text chunks to achieve retrieval precision and efficiency. This segment-wise approach is currently widely used in practice and is effective, especially when the context is self-contained within each segment. However, in domain-specific use cases such as electrical codes and standards, this approach may lead to significant context fragmentation. As an example, consider the following question asked of a database that stores the National Electrical Code (NEC):

*What is the minimum burial depth for direct-buried conductors under a parking lot?*

A retrieved chunk might contain the following response:

*The minimum cover depth shall be 24 inches for direct-buried conductors.*

While this statement appears relevant, it lacks critical contextual qualifiers. The retrieved chunk may omit the information that the requirement applies only for certain types of installations (for example, circuits that are rated 0 to 600 volts) and is located under certain locations (such as driveways or parking lots subject to vehicular traffic). Although the retrieval might be considered successful, the response output by the RAG pipeline might be incomplete or not follow the desired semantics if the installation condition or voltage rating is mentioned in a previous chunk. This points to a fundamental limitation of traditional chunk-based RAG systems: it is possible for each chunk to contain insufficient semantic or structural context to potentially support an in-depth, regulation-compliant answer. In more complex documents such as the NEC, where applicability is often determined by a combination of table values, conditional rules, and cross-referenced sections, such fragmentation can harm both the relevance of retrieved sections and the accuracy of a generative search. These limitations warrant looking for context-aware RAG approaches that try to keep and restore larger contextual windows during both retrieval and generation.

To address this, contextual RAG augments each chunk with its surrounding textual context (e.g., parent sections, headers, or preceding paragraphs) during embedding. This allows the retriever to maintain semantic continuity and structural fidelity. However, this comes at the cost of an increased token volume. Empirically, contextual RAG embeddings can require 2–3 times more tokens per chunk than traditional RAG approaches.

As token volume increases, careful model selection becomes essential, especially for real-time or cost-sensitive applications. For retrieval and summarization tasks that do not require advanced reasoning, it was found to be preferable to select lightweight LLMs with:

- High token throughput (e.g., 200–300 tokens/s),
- Low per-token cost (e.g., <$0.10 per input million tokens), and.
- Extended context windows (e.g., 1 million tokens).

These characteristics, summarized in Table 4, ensure that contextual RAG systems remain responsive and scalable, even as embedding and inference loads grow with richer document representations. At the time of writing the paper, Llama 4 Scout 17B 16E fulfilled these criteria and was used for the subsequent demonstrations of contextual RAG; however, given the rapid developmental pace in this domain, researchers should look for similar lightweight LLMs that might better align with the preferred characteristics listed in Table 4.

**Table 4:** A comparison between traditional vs. novel contextual RAG chunking strategies, highlighting trade-offs.

RAG strategy	Avg token per chunk (or chunk + context)	Retrieval fidelity	Embedding cost
Traditional RAG	~250–350 tokens	Medium	Low
Contextual RAG	~600–1,000 tokens	High	Higher (unless models like Gemini 2.5 Flash are used, with low $/million token cost)

**Figure 5:**
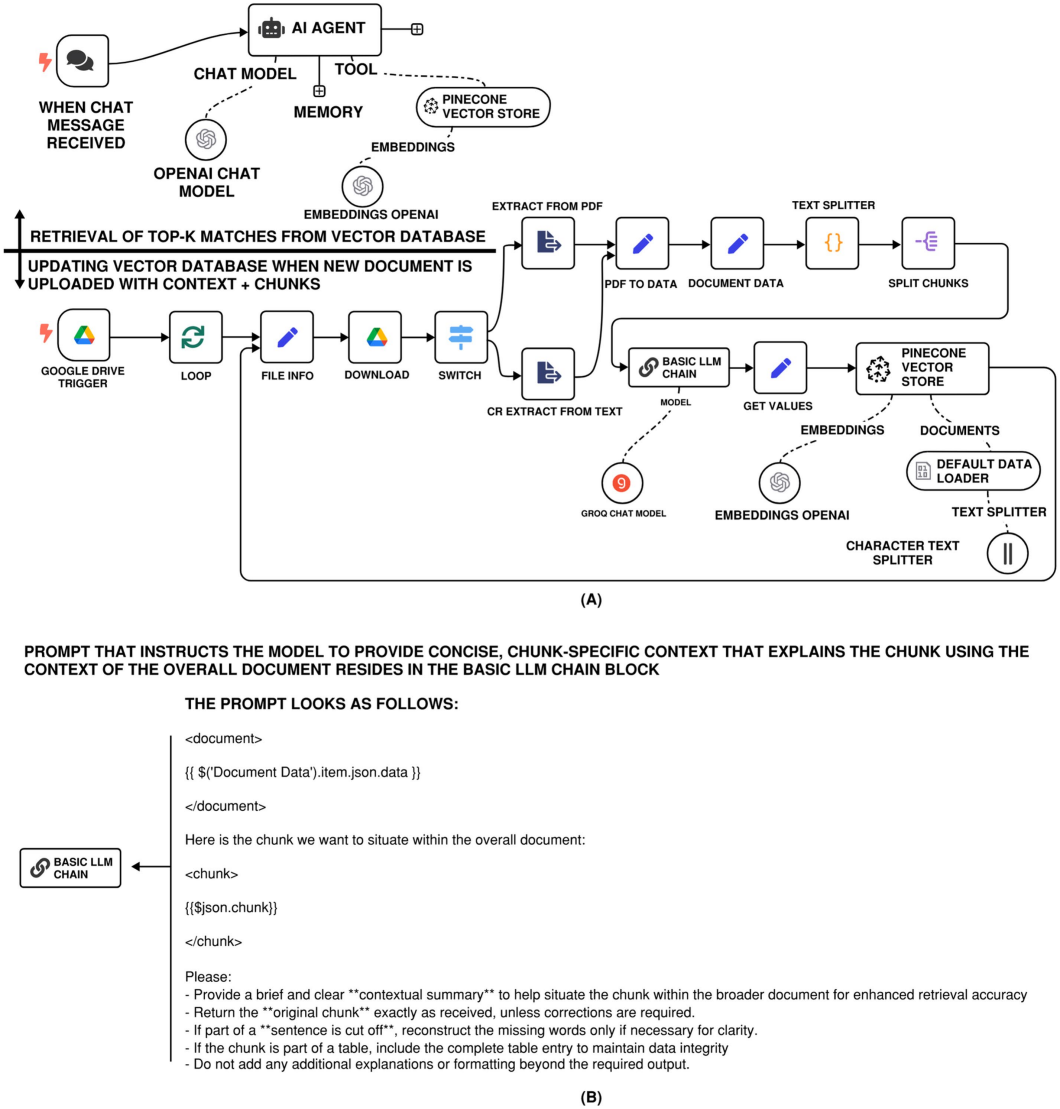
**(A)** n8n RAG-LLM workflow with contextual retrieval system allowing for prepending chunk-specific explanatory context to each chunk before embedding. **(B)** Prompt that instructs the model to provide concise, chunk-specific context that explains the chunk using the context of the overall document.

From a system architecture standpoint, the implementation of contextual RAG within an n8n workflow closely mirrors that of traditional RAG pipelines; recall Figure 3. The overall structure, as illustrated in Figure 5A, comprises document ingestion, embedding generation, vector storage, retrieval, and language model invocation, and remains fundamentally the same. However, contextual RAG introduces two key enhancements that differentiate it in both design philosophy and execution:

1. First, the text splitting and chunking were also done more deliberately, with explicit control on the size, overlap, and structural boundaries of the chunks. This splitting/chunking strategy ensures that semantically cohesive units, for example, complete table entries, complete regulatory exceptions, or paragraph-level logical constructs, are preserved as individual retrieval units. This refinement is essential for preserving the context that is valuable for the downstream retrieval and reasoning.
2. Second, instead of aggregating top-K retrievals into a single prompt, each chunk is individually passed to a “Basic LLM Chain” node within n8n. This node is configured with a well-crafted, structured prompt (user message), Figure 5B, that guides the LLM in evaluating each chunk’s relevance and factual contribution to the original query. The design of this prompt is inspired by prompt templates found in contextual RAG applications, such as those published by Anthropic (2024). The chain then filters or ranks responses from multiple chunks before synthesizing a final answer.

The working of the contextual retrieval system is tested by comparing the responses of both the traditional and contextual retrieval systems to questions that would require the system to refer to tabular data or exceptions to code sections in the NEC, and these results are shown in Table 5. The questions in Table 5 were framed through a collaborative series of discussions with practitioner engineers familiar with electrical codes and standards. As such, for the design of the experiment, the practitioner engineers had the following responsibilities:

- Ensuring the queries being structured are technically correct and align with NEC 2017,
- Ensuring that the queries being compiled are a fair representative sample covering all chapters of the NEC,
- Developing the ground truth for evaluation purposes.

**Table 5:** Validation of traditional versus contextual RAG-LLM responses using n8n workflows on query tasks based on the 2017 National Electrical Code (NEC).

Q&A responses and scores with traditional RAG-LLM	Q&A responses and scores with contextual RAG-LLM
**Q1. Prompt:** Based on the NEC, for solar photovoltaic (PV) system application, I want to know what is the correction factor that needs to be applied to PV cables for an ambient temperature of 55 °C. The temperature rating of the conductor being 75 °C rated. **A1. Ground truth:** According to NEC Table 690.31(A), the applicable correction factor is 0.67. **A1. Actual output from traditional RAG:** According to the National Electrical Code (NEC) 2017, specifically Table 310.15(B)(2)(a) (renumbered in later versions as Table 310.15(B)(1) or Table 310.15(B)(1) Adjustment Factors), the required correction factor is 0.70 for 75 °C rated PV conductors in 55 °C ambient conditions. **Score: 0.1981536822161493** **Reason:** The actual output yields a correction factor of 0.70 derived from a distinct NEC table, whereas the expected output indicates a correction factor of 0.67 from Table 690.31(A). This signifies a factual inaccuracy and a contradiction with the anticipated product. The real result fails to include the precise reference to Table 690.31(A), which is crucial information from the expected output.	**Q1. Prompt:** Based on the NEC, for solar photovoltaic (PV) system application, what is the correction factor that needs to be applied to PV cables for an ambient temperature of 55 °C, and the temperature rating of the conductor being 75 °C rated. **A1. Ground truth:** According to NEC Table 690.31(A), the applicable correction factor is 0.67. **A1. Actual output from contextual RAG:** According to NEC Table 690.31(A), for a 75 °C rated conductor in an ambient temperature of 51–55 °C, the applicable correction factor is 0.67. **Score: 0.8918686352485045** **Reason:** The output is factually true and presents the correct adjustment factor of 0.67, as anticipated. It comprehensively handles the anticipated output by delineating the NEC table and the temperature range, thereby enhancing clarity. The output is consistent and conforms to the context and specifications of the input. The sole small shortcoming is the rather excessive elaboration; however, it does not undermine the accuracy. **Score improved over traditional RAG implementation.**
**Q2. Prompt:** Could you please tell me the correction factor for a four current-carrying conductor based on NEC section 310? **A2. Ground Truth:** According to NEC Table 310.15(B)(3)(a), the applicable correction factor is 80%. **A2. Actual output from traditional RAG:** According to Section 310.15(3)(a) of the National Electrical Code (NEC), the adjustment factors for four current-carrying conductors are 1 and 2%. These factors are used to adjust the load diversity of the installation. **Score: 0.08819886431169416** **Reason:** The actual output erroneously designates the adjustment factors as 1 and 2%, which is inconsistent with the Expected Output’s 80% correction factor. It incorrectly cites section 310.15(3)(a) instead of Table 310.15(B)(3)(a). This illustrates factual flaws and inconsistencies with the expected output.	**Q2. Prompt:** Based on NEC section 310, what is the adjustment factor for a 4 current-carrying conductor? **A2. Ground truth:** According to NEC Table 310.15(B)(3)(a), the applicable correction factor is 80%. **A2. Actual output from contextual RAG:** According to the National Electrical Code (NEC) 2017, specifically Table 310.15(B)(3)(a), the applicable correction factor, as adjusted for ambient temperature if necessary, is 80%. **Score: 0.7562603783294554** **Reason:** The output accurately designates the adjustment factor as 80% in accordance with NEC Table 310.15(B)(3)(a), consistent with the anticipated outcome. However, it introduces extraneous information on ambient temperature adjustment, which is neither required by the input nor anticipated in the output, so it slightly diverges from the predicted response. **Score improved over traditional RAG implementation.**
**Q3. Prompt:** Based on section 310.10(H) of the NEC, if I have two parallel #8 AWG conductors, what is my restriction on overcurrent protection? **A3. Ground truth:** The system’s overcurrent protection for two parallel #8 AWG conductors must be built to prevent each conductor’s ampacity from being exceeded in the event that one or more of the parallel conductors are inadvertently disconnected. **A3. Actual output from traditional RAG:** Section 310.10(H) of the NEC permits parallel conductors in raceways, multiconductor cables, or direct-buried single-conductor cables, provided they satisfy specific standards. The cumulative current-carrying capacity of the parallel circuits must not surpass the ampacity rating of the raceway or overhead circuit. If the ungrounded service-entrance conductors are installed in parallel, they must be scaled to correspond with the ampacity and rated voltage of the ungrounded service-entrance wires. In the provided context, it is crucial to ensure that the combined current-carrying capability of two parallel #8 AWG conductors does not surpass the ampacity rating of the raceway or overhead circuit. **Score: 0.2610665811531422** **Reason:** The actual result does not explicitly address the restriction on overcurrent protection concerning the scenario in which one or more parallel conductors become disconnected, a crucial element of the anticipated output.	**Q3. Prompt:** Based on section 310.10(H) of the NEC, if I have two parallel #8 AWG conductors, what is my restriction on overcurrent protection? **A3. Ground truth:** With two parallel #8 AWG conductors, the overcurrent protection should be such that the ampacity of individual conductors will not be exceeded if one or more of the parallel conductors become inadvertently disconnected. **A3. Actual output from contextual RAG:** NEC 2017, Section 310.10(H) says that for two parallel #8 AWG conductors, each one must be safeguarded so that no one exceeds its ampacity. **Score: 0.8042020327293316** **Reason:** The actual output aligns well with the expected output by stating that each conductor must be protected to not exceed its ampacity, which is consistent with the requirement that the ampacity of individual conductors should not be exceeded. **Score improved over traditional RAG implementation.**

Score is computed using DeepEval’s correctness with the criteria [GEval] metric.

^*^For this implementation, a chunk size of 2,000 was used for both traditional RAG setup and contextual RAG setup, with an overlap of 20.

The median and interquartile range (IQR) of the performance scoring for contextual and traditional RAG were found to be 0.83 [0.79, 0.89] and 0.62 [0.26, 0.77], respectively. To determine whether there was a significant improvement in the performance of contextual RAG over the traditional RAG, we decided to conduct a one-sided paired Wilcoxon Signed test. The choice of the Wilcoxon Signed test was dictated by the non-normal distribution of the observed scores for the traditional RAG. Considering a significance level of 0.05, the resulting Wilcoxon test reported a *p*-value of <0.0001, indicating the scores from contextual RAG to be statistically greater than those of the traditional RAG.

While contextual RAG is a significant improvement over traditional RAG-LLM (or LLMs on their own) in terms of retrieval fidelity, both traditional and contextual RAG suffer from hallucination, especially in the following situations:

1. Complex or multi-layered prompts, when the question requires reasoning across several conditions or sentences.
2. Cross-referenced rules are ubiquitous in engineering and regulatory documents. One area of the document refers to definitions, exceptions, or constraints in another section.

In such instances, the language model is capable of generating plausible, yet semantically distorted responses, even if the correct passage is partially retrieved. This issue arises because the generative component of the pipeline still relies on learned statistical patterns and does not inherently verify or enforce rule-bound reasoning.

Therefore, rather than being considered as final sources of truth, both traditional and contextual RAG should be viewed as instruments to speed up the search and localization of domain-specific information. The final output must always be checked against the original text from the authoritative source, whether it be a technical handbook, regulatory code, or engineering standard, even if these systems greatly lessen the cognitive and temporal strain of manual technical engineering code-book navigation. In high-assurance fields where precision, safety, and regulatory compliance are non-negotiable, this verification phase is nevertheless crucial.

## 6 State-of-the-art advanced implementation with multi-brain knowledge graph–based RAG

Knowledge graphs (Ji et al., 2022a; Peng et al., 2023; Tiwari et al., 2021) provide a superior approach for modeling relationships between concepts by structuring information, including entities and their interconnected associations. While vector databases use numerical proximity in an embedding space to show semantic similarity, knowledge graphs use a graph-based design to keep obvious, understandable relationships, like hierarchical, causal, or functional connections. Between vector RAG systems and knowledge graph-based RAG systems, each has its own advantages and use case scenarios, and one is not a replacement for the other.

In a knowledge graph, each node represents a distinct concept or entity, and the edges illustrate the nature of their interaction, enabling advanced reasoning, ontology-based inference (Baclawski et al., 2017), and contextual elucidation. Neo4j shows that graph databases are the basic building blocks that make it possible for users to run complex queries using graph traversal or SPARQL-like languages. This graph style is highly useful in sectors where there are obvious paths for making decisions, rules-based logic, or where explainability and relationship integrity are very important. Knowledge graphs not only have clear and easy-to-understand structures, but they also have unique analytical benefits that go beyond those of typical embedding-based representations. These affordances make it easier to find hidden patterns, undertake exploratory research, and look into nuanced linkages across complex information domains:

1. *Identification of structural gaps and blind spots:* Knowledge graphs, as a visual and relational depiction of information, can reveal under-connected or isolated nodes, which may signify neglected or poorly integrated concepts. These blind spots can be utilized to produce innovative discoveries by encouraging new, contextually pertinent linkages between dissimilar concepts.
2. *Exploratory pathways for idea navigation:* Knowledge graphs facilitate intuitive exploration among concepts. By using the connectivity of a node inside the graph, users might uncover indirect yet substantial relationships between concepts and hypotheses, perhaps leading to interdisciplinary discoveries or revisions of conceptual frameworks.
3. *Revealing nuance through concept removal:* By algorithmically or manually removing dominant or highly connected nodes, knowledge graphs can surface latent structures and peripheral relationships. This technique brings to the forefront more subtle, contextually rich ideas that are crowded out by the prevailing big picture and thus supports a deeper interpretation and nuanced understanding of the information space.

**Figure 6:**
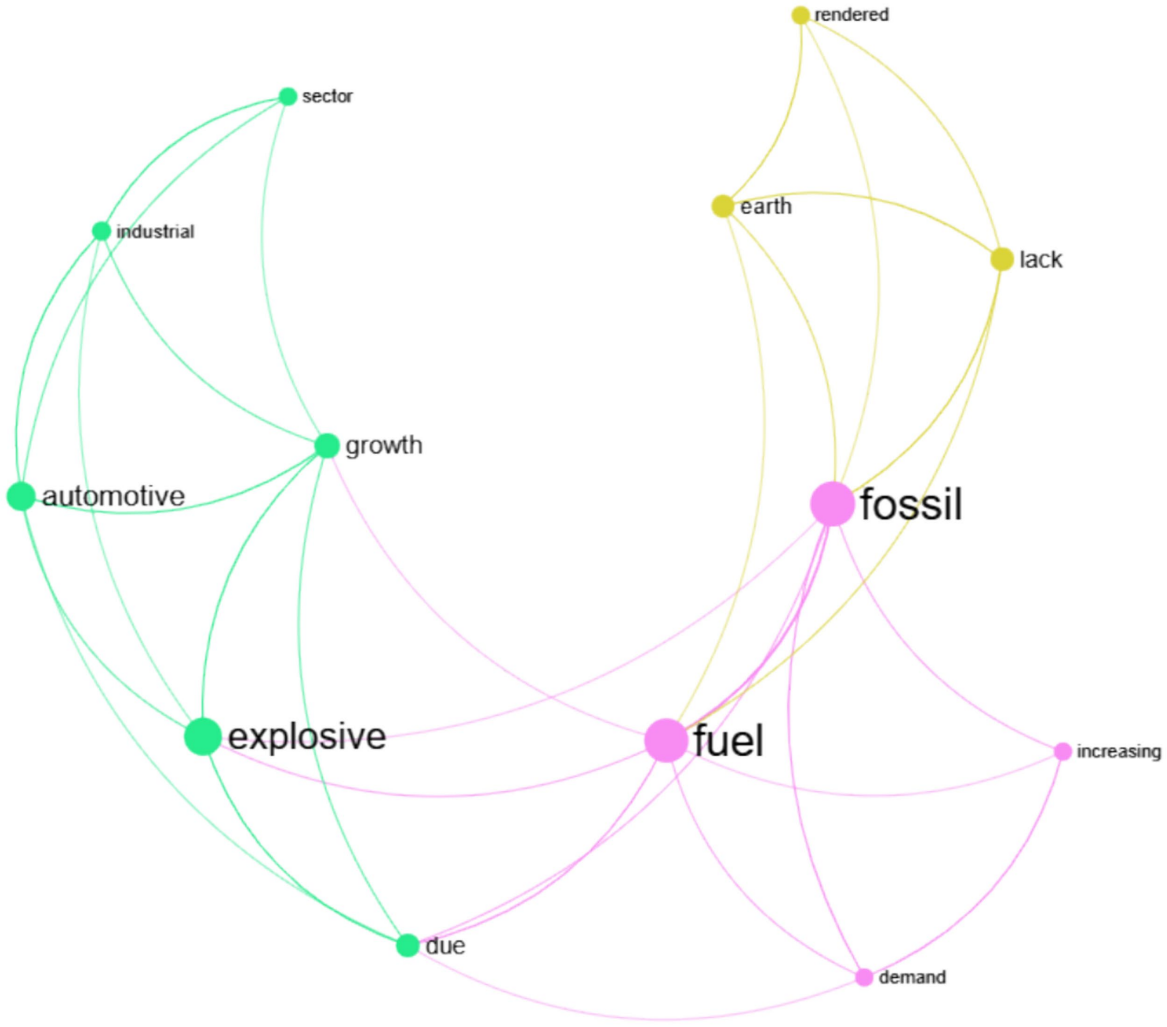
A simple knowledge graph with the clustering of similar nodes based on the Louvain community detection algorithm. Each cluster is assigned a unique color.

In the relation extraction process, semantic relationships are first identified from unstructured text and mapped as edges connecting concept nodes in the knowledge graph (Ji et al., 2022b). The graph is then analyzed using the Louvain community detection algorithm (Blondel et al., 2008) from network science, which clusters densely connected nodes together. Nodes belonging to the same community are assigned the same color, visually revealing meaningful relational groupings and latent structure within the extracted knowledge. Figure 6 shows such a knowledge graph for the following simplified example: “*The ever-increasing demand for fossil fuels due to explosive growth in automotive and other industrial sectors has rendered the earth lacking fossil fuels.*”

When extended to real-life text-rich applications, knowledge graphs offer powerful capabilities for representing structured knowledge, and these can be combined with an intelligent AI agent inside a knowledge-graph–driven Retrieval-Augmented Generation (RAG) framework. In this setup, the agent does not follow a single linear path but instead navigates across one or more linked knowledge graphs depending on the input prompt. Using the graph structure to guide retrieval, the system is able to capture semantically rich context, highlight hidden connections, and generate answers that are both precise and easier to interpret because they remain tied to structured domain knowledge. Such an approach is particularly useful in engineering research, where overlapping and interdependent domains require contextual representation. To illustrate the concept, three separate knowledge graphs were built, each organized around a specific theme. In this case, the thematic focus was an electrical engineering challenge: enhancing grid resiliency by drawing on both the researcher’s own work and technical reference materials published through IEEE and CIGRE. For demonstration purposes, these three custom-built knowledge graphs, labeled brain 1, brain 2, and brain 3, were created through a manual curation process leveraging the authors’ domain knowledge to support the construction of these knowledge graphs. For more advanced applications, an AI agent can be leveraged to curate the necessary manuscripts before passing them into a knowledge graph for processing. The composition level details for brain 1, brain 2, and brain 3 are as follows:

1. Brain #1 contains the authors’ own body of work, including prior publications (Ghosh and Suryawanshi, 2024; Ghosh and Dutta, 2020) and internal reports addressing topics such as grid maintenance strategies, resilience measurement, substation floor layout, and fire hazard evaluation.
2. Brain #2 is built from IEEE resources, including technical reports like IEEE TR83 (Bose et al., 2020) and selected papers (Safdarian et al., 2024; Bhattarai et al., 2022; Tabassum et al., 2024) from the IEEE Xplore Digital Library. The IEEE resources focus on technical frameworks, analytical tools, and performance indicators that support resilience.
3. Brain #3 draws on selected CIGRE technical reports (Ciapessoni et al., 2023) and related manuscripts (Itotani et al., 2024), and assembled to provide expertise on sustainable practices for strengthening substation reliability. This includes the integration of environmentally friendly technologies and the use of remote maintenance approaches.

**Figure 7:**
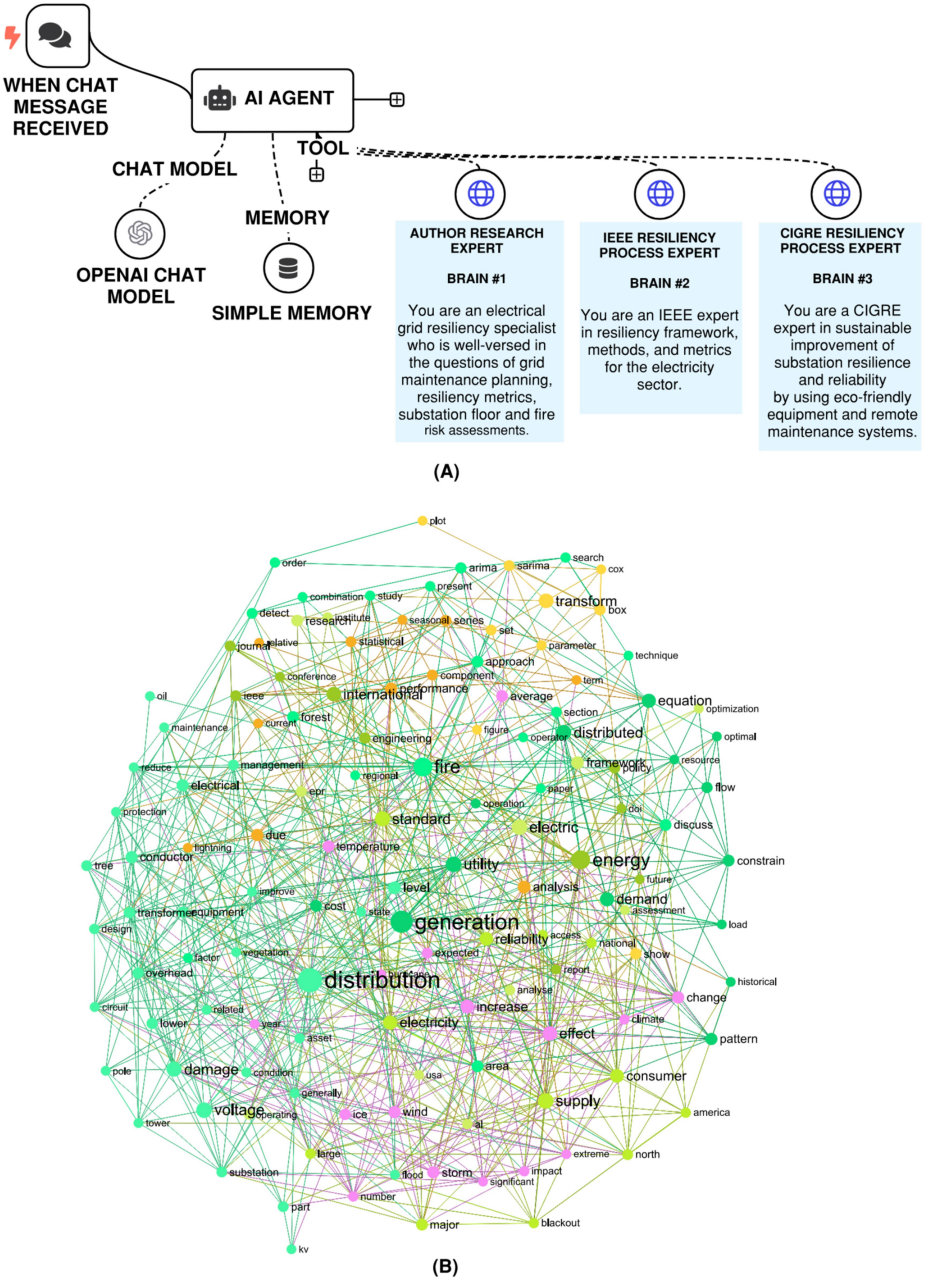
**(A)** n8n RAG-LLM workflow with multiple custom knowledge graphs, each housing unique knowledge. **(B)** A visualization of the ‘author research expert’ (brain 1) knowledge graph. Similar nodes are clustered together based on the Louvain community detection, and each of these clusters is assigned a unique color.

The structure of the knowledge graph–based RAG system is shown in Figure 7A, with all three knowledge graphs (brain 1, 2, and 3) being created and deployed using the InfraNodus platform (Paranyushkin, 2024). Figure 7B highlights one of these graphs as an example. Much like earlier versions of traditional and contextual RAG, this implementation was built with the n8n platform and has been made accessible for external use, as noted in the data availability section. Depending on the detail and scope of a given research query, the AI agent may consult a single graph, multiple graphs, or all three interconnected “brains” to capture the most relevant context and expand the depth of retrieved knowledge. Examples of these queries, along with the corresponding graph-driven outputs, are provided in Table 6. As shown, the agent actively decides which graph or combination of graphs to draw from in order to refine its responses.

**Table 6:** Testing the working mechanism of the knowledge graph-based RAG implementation with the AI agent intelligently tapping the necessary dynamic knowledge graphs (brains).

Prompt into knowledge graph-based RAG	Brains that the AI agent consulted	Response from the knowledge graph-based RAG agent
**Q1. Prompt:** Referring to the IEEE technical reference documents and the author’s own research, can you summarize very briefly how fast trip relay settings can help in high fire threat areas.	Brain 1 and Brain 2 (see Figure 7A)	**A1. Actual output from knowledge graph-based RAG agent:** Fast-trip relay configurations are particularly advantageous for power systems functioning in areas susceptible to wildfires. Research and IEEE publications indicate that these configurations assist in several essential aspects. They facilitate the prompt identification and resolution of faults on a line. Prompt isolation prevents a minor disturbance from escalating into a significant failure that could ignite a fire. In addition, by decreasing the detection threshold, relays act as a preventative measure, diminishing the likelihood of an electrical fault becoming an ignition source. This method not only mitigates fire danger but also enhances overall reliability by maintaining power continuity and diminishing the probability of outages that could hinder firefighting operations. In summary, implementing fast-trip settings serves as a focused preventive strategy and a pragmatic dependability measure in high-risk zones.
**Q2. Prompt:** Referring to CIGRE documents and the author’s own research, can you list briefly what are the benefits of the SF6 alternative circuit breaker?	Brain 1 and Brain 3 (see Figure 7A)	**A2. Actual output from knowledge graph-based RAG agent:** Vacuum and synthetic-air circuit breakers offer clear advantages over SF₆ types. The biggest difference is seen to be in the environmental aspect, given that SF₆ is a potent greenhouse gas, while these alternatives eliminate that problem entirely. Handling is also simpler as operators do not need to deal with gas recovery systems or think about accidental release of potent greenhouse gases. Maintenance costs are also lower as a result. Additionally, vacuum breakers, for example, can run for long periods without service, which reduces expenses and downtime. Together, these factors make SF₆-free breakers a practical choice, especially where sustainability targets and safety requirements are becoming stricter.

An interesting question that may arise here is how the AI agent intelligently selects which knowledge graph(s) to query? In essence, during the construction of the AI agents that tap into the different knowledge graphs, an Infranodus compliant system message is added within the AI agent as follows, which holds to key to the method’s intelligence: “*Always use either EightOS or Polysingularity tool before sending a response to the model. You have to use at least one of them, the one that think is more suitable. Or both or all if both or all can provide some help.*”

This system message gives the AI agent the necessary context, and as each graph is described, it will know when to choose it, depending on what the user has defined.

**Figure 8:**
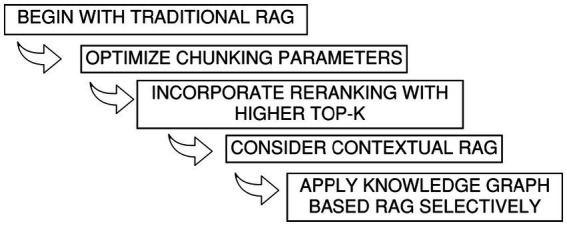
Illustration shows a multi-step guide to reduce API usage for RAG implementation.

Based on a series of prompting interactions with the knowledge graph-based RAG system, the following conclusions were made:

1. *Higher API usage cost for knowledge graph generation:* In practice, building a knowledge graph from materials such as PDFs or structured documents uses far more API resources than simply loading text into a vector database. A standard embedding job might use only one or two tokens for each word, but graph generation is different. When relation extraction, entity resolution, or ontology alignment are involved, token use can rise to five to ten times higher for the same document. The extra cost comes from the heavier semantic parsing and the need to map relationships rather than just storing word vectors.
2. *Recommendation to reduce API usage:* Figure 8 presents a staged approach for rolling out RAG systems with cost in mind. The idea is to begin with conventional setups and only move toward contextual or knowledge-graph–driven RAG when the task at hand truly demands stronger semantic depth or reasoning across domain-specific relationships.
3. *Limitations with numerical tabular data:* Knowledge graphs are a poor fit for dense numerical tables since their strength lies in capturing concepts and relationships, not large matrix-style datasets. Applying them to standards with heavy tabular content, like the NEC or NFPA documents, ends up being restrictive. In practice, forcing numerical tables into a graph strips out detail and makes retrieval clumsy. Row-to-row dependencies, column statistics, and numeric precision do not map cleanly into entity–relation triples, so the ability to run meaningful graph-based inference is reduced.
4. *Expanded exploratory potential with multiple knowledge graphs:* One primary advantage of implementing many domain-specific knowledge graphs is that it expands the range of information available to the AI system. The agent can traverse one or more graphs simultaneously, based upon the requirements of the query. This enables the identification of relationships that may not be evident in a singular graph. Consequently, it facilitates more exploratory analysis and thereby aids users in tracking concepts across interconnected domains. This can often reveal obscured connections that may inspire novel avenues of study.
5. *Other observations:* Some additional observations that were made are docketed as follows:

- In a traditional vector-based RAG setup, there is a loss of structural information when chunking documents, while such hierarchies and relationships are preserved in a knowledge graph-RAG setup.
- Knowledge graph-RAG was better able to support complex reasoning across multiple facts, such as exceptions to certain engineering code sections, or supplementary discussions on a particular subject within a research paper.
- While both contextual RAG and knowledge graph–based RAG aim to improve retrieval-augmented generation, they are architecturally and functionally optimized for different types of information retrieval tasks. Contextual RAG excels at retrieving and preserving semantic continuity from unstructured text, making it particularly effective for answering questions that rely on narrative explanations or rule statements embedded in paragraphs (e.g., code clauses or installation descriptions). In contrast, KG-RAG is optimized for relational reasoning and structured knowledge traversal, making it well-suited for queries that involve interdependence, multi-node relationships, or exception handling.

6. *Author’s recommendation:* As a user, one should make a predetermined assessment as to what RAG structure might work best for their particular use case.

## 7 Summary and future work

This paper shows that modern automation platforms like n8n make it possible to set up Retrieval-Augmented Generation (RAG) pipelines with very little coding. Engineers and researchers can use these tools to create working RAG systems that pull and process domain-specific material with ease. Depending on the application, repositories can be indexed either through vector databases or knowledge graphs, giving users flexible options for context-aware retrieval. Vector-based RAG remains the more common choice because of its efficiency and simple deployment, but knowledge-graph approaches provide stronger semantic structure and clearer interpretability. That makes them especially useful when navigating the dense interconnections found in engineering standards.

At the moment, large-scale use of graph-augmented RAG is held back by higher computational demands and API costs linked to graph construction and reasoning. As these costs drop and APIs improve, knowledge-graph retrieval is expected to grow in adoption and could eventually outperform vector search in precision and domain relevance. This pathway is particularly important in electrical engineering, where understanding how technical terms, clauses, and procedures relate to one another is critical. The combination of n8n with tools like Infranodus already demonstrates that this direction is feasible and practical for document-heavy environments.

Looking ahead, several areas offer opportunities for further development. These include:

1. *Multimodal retrieval capabilities:* A promising direction for future work is the integration of multimodal retrieval capabilities into contextual and knowledge graph RAG systems. In engineering, valuable information is not limited to text but is often embedded in schematics, drawings, tables, and other structured resources. Future RAG workflows should be able to extract relationships from these non-textual artifacts, associate them with textual knowledge, and represent them within a unified graph structure. For example, a retrieval system could pair a relay’s specification sheet with the relevant section of a one-line diagram to answer technical queries with greater accuracy. This kind of integrated retrieval would allow reasoning over multiple formats at once and significantly enhance practical utility in engineering applications,
2. *Improving chunking and indexing strategies:* Another important area of development involves improving chunking and indexing strategies, particularly when dealing with dense numerical tables that are common in engineering standards and equipment specifications. Traditional chunking methods often treat these tables as plain text, which strips away their structural meaning and makes retrieval inefficient. Large tables can consume excessive tokens without preserving the underlying relationships between rows, columns, and units. A more structure-aware chunking approach would preserve table headers, normalize units, and index individual cells or logical table sections, allowing the system to retrieve only the relevant values rather than entire tables. This targeted strategy would reduce computational cost and significantly improve precision when responding to engineering queries that rely on numerical data,
3. *Real-time feedback mechanisms:* Future research should also explore real-time feedback mechanisms that allow RAG systems to learn from expert users. Engineering work is inherently iterative, and domain experts can quickly identify gaps or inaccuracies in system outputs. By incorporating features that let users tag, approve, or correct specific pieces of information, RAG systems can refine their retrieval models over time. This type of closed feedback loop would lead to higher precision and reduce the amount of human effort required to verify answers, resulting in more dependable system performance in real engineering workflows., and.
4. *Expanding multilingual capabilities:* Finally, expanding multilingual capabilities will be essential to make RAG systems more robust and globally relevant. Engineering standards and operational manuals are often published in multiple languages, and collaborative projects frequently involve international teams.
5. *Hybrid RAG architecture:* Another promising direction for future research is the development of a hybrid RAG architecture that balances performance with cost efficiency. Knowledge graph–based RAG (KG) approaches provide strong contextual reasoning but are typically associated with significantly higher API and computational costs compared to vector-based methods. To address this, a combined strategy can be explored in which KG RAG is selectively applied to complex, reasoning-heavy queries, while VectorRAG handles more direct or fact-based retrieval tasks.

## Data Availability

The n8n templates generated as part of this manuscript can be accessed on the project’s GitHub page: https://github.com/sghosh27/Low-Code-RAG-LLM-Framework-for-Context-Aware-Querying-in-Electrical-Standards-Design-and-Research along with Python code for document parsing. A Python notebook is also available for scoring LLM generated outputs versus ground truth.
